# Aetiological overlap between anxiety and attention deficit hyperactivity symptom dimensions in adolescence

**DOI:** 10.1111/jcpp.12318

**Published:** 2014-09-08

**Authors:** Giorgia Michelini, Thalia C. Eley, Alice M. Gregory, Tom A. McAdams

**Affiliations:** ^1^ MRC Social Genetic and Developmental Psychiatry Centre Institute of Psychiatry King's College London London UK; ^2^ Department of Psychology Goldsmiths University of London London UK

**Keywords:** Anxiety, ADH problems, genetics, twins, adolescence

## Abstract

**Background:**

Anxiety and attention‐deficit/hyperactivity (ADH) problems are common in adolescence, often co‐occur, and are characterised by high heterogeneity in their phenotypic expressions. Although it is known that anxiety and ADH problems correlate, the relationships between subtypes of anxiety and ADH problems have been scarcely investigated.

**Methods:**

Using a large population sample of adolescent twins and siblings we explored the phenotypic and aetiological association between anxiety subtypes (panic/agoraphobia, separation anxiety, social anxiety, physical injury fears, obsessive‐compulsive symptoms and generalised anxiety) and the two ADH dimensions (attention problems and hyperactivity/impulsivity). Both phenotypes were assessed using self‐report questionnaires.

**Results:**

The association between ADH problems and anxiety could be entirely attributed to attention problems, not hyperactivity/impulsivity. Most of the correlations between anxiety subtypes and attention problems showed an approximately equal role of genetic and nonshared environmental factors.

**Conclusions:**

The high heterogeneity within anxiety and ADH problems should be taken into account in order to better understand comorbidity between them.

## Introduction

Anxiety disorders and attention‐deficit/hyperactivity disorder (ADHD) are among the most common psychiatric disorders (Ford, Goodman, & Meltzer, 2003; Kessler et al., 2005). Both conditions often onset in childhood or adolescence (Polanczyk et al., 2010; Ramsawh, Chavira, & Stein, 2010), show high continuity into adulthood (Gregory et al., 2007; Kieling et al., 2010), and severely impact many aspects of individual's lives (Asherson, 2005; Gregory et al., 2007).

Both anxiety disorders and ADHD frequently co‐occur with other disorders. For example, anxiety is commonly associated with depression (Zavos, Rijsdijk, Gregory, & Eley, 2010) and ADHD with disruptive behaviour disorders (Biederman, Newcorn, & Sprich, 1991). Anxiety and ADHD also co‐occur. Clinical and epidemiological studies report rates of co‐occurrence between anxiety and ADHD as being higher than 25% (Biederman et al., 1991; Bowen, Chavira, Bailey, Stein, & Stein, 2008).

Heterogeneity is a common feature of many psychiatric conditions (Bagdy & Juhasz, 2013), including anxiety and ADHD. Anxiety is often disaggregated into subtypes such as those included in the Spence Children's Anxiety Scale (SCAS, McLaughlin, Stewart, & Taylor, 2007; Spence, 1997): panic/agoraphobia, separation anxiety, social anxiety, physical injury fears, obsessive‐compulsive symptoms and generalised anxiety. The heterogeneity of anxiety has also been highlighted in the DSM‐5, by mapping anxiety subtypes onto three broad categories: anxiety disorders; obsessive‐compulsive and related disorder; and trauma‐ and stressor‐related disorders (American Psychiatric Association [APA], 2013). Twin studies, which are able to decompose covariance between traits into that attributable to genes and environment (Rijsdijk & Sham, 2002), support the notion of anxiety as a partially heterogeneous construct, demonstrating aetiological heterogeneity (as well as overlap) across anxiety subtypes (Eley et al., 2003; Lau, Gregory, Goldwin, Pine, & Eley, 2007). ADHD, although a single disorder, is defined by two core symptom dimensions of inattention and hyperactivity/impulsivity (APA, 2013). Individuals with ADHD can differ in the extent to which they display symptoms from each of these dimensions. This is reflected in the DSM‐5 by the definition of 3 ADHD presentations: predominantly inattentive (ADHD‐I), predominantly hyperactive‐impulsive (ADHD‐H) and combined (ADHD‐C) presentations (American Psychiatric Association, 2013). Twin studies report significant genetic heterogeneity, as well as genetic overlap, between attention‐deficit/hyperactivity (ADH) dimensions in childhood and adolescence (Greven, Rijsdijk, & Plomin, 2011; McLoughlin, Ronald, Kuntsi, Asherson, & Plomin, 2007).

To date, few studies have explored whether inattention and hyperactivity/impulsivity may be differentially associated with anxiety. Some evidence suggests that anxiety is associated with reduced hyperactivity/impulsivity in ADHD youth (Epstein, Conners, Erhardt, March, & Swanson, 1997; Pliszka, 1992), which might suggest that co‐occurrence between ADHD and anxiety is attributable to a relationship between inattention and anxiety rather than hyperactivity/impulsivity and anxiety. Furthermore, ADH dimensions may be differentially associated with anxiety subtypes (Lahey et al., 2008), with initial evidence suggesting that inattention may be more strongly linked to some forms of anxiety, such as withdrawal/social anxiety and generalised anxiety, than is hyperactivity/impulsivity (Gadow & Sprafkin, 1998; Lahey et al., 2008; Willcutt et al., 2012). One explanation for the relationship between inattention in ADHD and anxiety might be found in the role of attention in anxiety disorders (Beck & Clark, 1997). Attention biases and difficulty in switching attention from a threat stimulus to another stimulus are key elements in the aetiopathogenesis of anxiety disorders (Cisler & Koster, 2010), and might produce enhanced inattention to everyday activities in anxious individuals. However, as recently pointed out by a meta‐analysis of ADH dimensions, research into the association between ADH dimensions and anxiety subtypes has so far yielded mixed results and warrants further studies (Willcutt et al., 2012). Similarly, the genetic and environmental influences on their overlap have barely been explored. Only one twin study has examined the association between attention problems and internalising problems: in a child sample, Schmitz and Mrazek (2001) found substantial genetic overlap underlying the association between attention problems and the ‘anxious/depressed’ and ‘withdrawn’ scales of the Child Behaviour Checklist (CBCL; Achenbach, 1991). Shared and nonshared environment correlations were moderate and small respectively. Schmitz and Mrazek (2001) did not disaggregate ADH problems and anxiety into dimensions, so it remains unclear how the heterogeneity of ADHD and/or anxiety may affect their aetiological overlap.

In the present study, we examined the association between ADH dimensions (attention problems and hyperactivity/impulsivity) and anxiety subtypes (panic/agoraphobia, separation anxiety, social anxiety, physical injury fears, obsessive‐compulsive symptoms and generalised anxiety symptoms; Spence, 1997), both measured with self‐report ratings in a sample of adolescent twins and siblings. Since both ADHD and many forms of anxiety are often first diagnosed during adolescence (Asherson, 2005; Gregory et al., 2007), studying their association in this developmental period is crucial. In particular, we predicted that attention problems would be more strongly associated with anxiety subtypes than would hyperactivity/impulsivity. We also aimed to estimate the extent to which associations between ADH and anxiety subtypes were due to overlapping genetic and environmental influences, and whether the nature of this overlap differed between pairs of symptoms domains.

## Methods

### Participants

Data were drawn from the Genesis 12–19 (G1219) twin and sibling study, a longitudinal study of adolescent twins and siblings (ages 12–19 years at initial contact). This sample combines data from adolescent offspring of adults from a large‐scale population‐based study (GENESiS study, Sham et al., 2000) with a random selection of twins born between 1985 and 1988 identified in collaboration with the UK Office of National Statistics. Full details on the G1219 sample can be found elsewhere (McAdams et al., 2013). In the G1219 sample, levels of parental education were somewhat higher (39% educated to A‐level, corresponding to the highest level of school‐based education in the UK, or above) than in a nationally represented sample of parents where 32% were educated to A‐level or above (Meltzer, Gatward, Goodman, & Ford, 2000). We focus here on the third wave of the study (the only wave where both anxiety and ADH problems were measured).

Zygosity was established through a parental questionnaire assessing physical similarity between twins (Cohen, Dibble, Grawe, & Pollin, 1975). The present sample consisted of 88 MZ male, 134 MZ female, 64 DZ male, 130 DZ female, 214 opposite‐sex DZ twin pairs and 30 male, 51 female and 71 opposite‐sex sibling pairs (*n* = 1564). The mean age of the sample was 17 years (range 14–23, *SD* = 1.66). All participants aged 16 and over provided informed consent. For those under 16 years, informed consent was obtained from their parents/guardians. Ethical approval for the study was granted by the Research Ethics Committee of the Institute of Psychiatry and the South London and Maudsley NHS trust.

### Measures

#### *Anxiety*

Symptoms of anxiety were assessed using the Spence Children's Anxiety Scale (SCAS; Spence, 1997), a self‐report questionnaire developed to assess anxiety symptoms in children and adolescents. The SCAS comprises 44 items measured on a four‐point scale and generates six subscales: panic/agoraphobia, separation anxiety, social anxiety, physical injury fears, obsessive‐compulsive symptoms and generalised anxiety symptoms. In relation to the DSM‐5, most SCAS subscales reflect disorders under the ‘anxiety disorders’ category, whereas SCAS obsessive‐compulsive symptoms map onto the ‘obsessive‐compulsive and related disorder’ category. The internal consistency and the test–retest reliability of this measure are generally high (Spence, 1997). In the current study, the Cronbach's alphas of the subscales were .79 (panic/agoraphobia), .67 (separation anxiety symptoms), .76 (social anxiety), .50 (physical injury fears), .76 (obsessive‐compulsive symptoms) and .78 (generalised anxiety symptoms).

#### ADH problems

The G1219 study included questionnaire items from the YSR and YASR (Achenbach, 1991). By referring to the YSR/YASR ‘attention problems’ scales (Achenbach, 1991) and the YSR DSM‐oriented ADHD scale detailed in Achenbach and Rescorla (2001), we identified the following items assessing ADH problems: ‘I act too young for my age’, ‘I day‐dream a lot’, ‘I have trouble concentrating or paying attention’, ‘I talk too much’, ‘I am louder than others’, ‘I have trouble sitting still’, ‘I act without stopping to think’, ‘I fail to finish things I should do’, ‘My behaviour is irresponsible’, ‘I am too dependent’, ‘My school work or job performance is poor’.

### Analyses

#### Derivation of attention and hyperactivity/impulsivity problems dimensions

ADH problems were disaggregated into their symptom dimensions via an exploratory factor analysis (EFA; rotation method: varimax) of the YSR/YASR items measuring attention problems, hyperactivity and impulsivity on a random half of the sample (only one twin/sib of each pair). We employed EFA because the YSR/YASR was not specifically designed to distinguish attention from hyperactivity/impulsivity (originally there was an ‘attention problems’ scale, but the items assessing hyperactivity and impulsivity were not included; Achenbach, 1991; Achenbach & Rescorla, 2001). As hypothesised, our EFA yielded two factors with eigenvalues ≥1, with all items (apart from the cross‐loading ‘I act too young’ and ‘I act without stopping to think’ items) clearly loading on one of these two factors (see Table S1). The items loading on the first factor (‘I day‐dream a lot’, ‘I have trouble concentrating or paying attention’, ‘I fail to finish things I should do’, ‘My behaviour is irresponsible’, ‘I am too dependent’, ‘My school work or job performance is poor’) were consistent with the ‘attention problems’ scale of YSR/YASR, and thus an attention problems scale summing these items was created. The items loading on the second factor (‘I talk too much’, ‘I am louder than others’, ‘I have trouble sitting still’), all referring to symptoms of hyperactivity/impulsivity, were summed to create a hyperactivity/impulsivity scale. Cronbach's alpha coefficients were .70 and .66 for attention and hyperactivity/impulsivity problems respectively.

#### Phenotypic analyses

Sex and age effects were examined with linear regression analyses in Stata version 10.0 (StataCorporation, 2007) using the cluster command to account for the nonindependence of observations originating from within the same family (i.e. twin/sibling pairs).

To correct for positive‐skewed data, all variables were transformed using the rank‐based van der Waerden's transformation (Lehmann & D'Abrera, 1975). Phenotypic relationships between variables were explored using full and partial correlations with the structural‐equation modelling programme OpenMx (Boker et al., 2011). First, full correlations between anxiety scales and ADH scales were run. Second, partial correlations between anxiety scales and each ADH scale (controlling for the other) were run to explore whether one of the two ADH scales specifically drove the association with anxiety. For example, when exploring the association between attention problems and panic/agoraphobia, models controlled for hyperactivity/impulsivity.

#### Genetic analyses

The twin/sibling design involves comparing correlations between MZ twins, who share all genetic effects, with those of DZ twins and siblings, who share on average 50% of their segregating genes. Phenotypic variance can then be decomposed into that attributable to additive genetic effects (A), shared environment effects (C) which make members of a twin pair similar to one another, and nonshared environment effects (E) which make members of a twin pair differ, including error.

**Figure 1:**
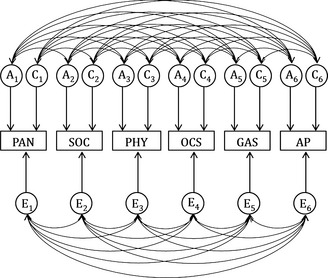
Full correlated factor solution for study variables. PAN, panic/agoraphobia; SOC, social anxiety; PHY, physical injury fears; OCS, obsessive‐compulsive symptoms; GAS, generalised anxiety symptoms; AP, attention problems. A_1_–A_6_, C_1_–C_6_ and E_1_–E_6_ respectively: additive genetic, shared environmental and nonshared environmental factors

Variables were sex‐ and age‐regressed prior to genetic analyses as is standard practise for quantitative genetic model‐fitting (McGue & Bouchard, 1984). Structural‐equation models were fitted in OpenMx using maximum likelihood estimation in eight zygosity groups (MZ male, MZ female, DZ male twins and siblings, DZ female twins and siblings, DZ opposite‐sex twins and siblings). A correlated factors solution (Figure 1) was fitted to the data, which assumes that each variable has unique A, C and E influences, which correlate with the A, C and E influences affecting other variables. Genetic, shared environmental, and nonshared environmental correlations provide an indication of the degree of overlap between aetiological influences on variables (Rijsdijk & Sham, 2002). Information about the precision of parameter estimates was obtained by likelihood‐based confidence intervals (CIs). The Akaike information criterion (AIC) and *χ* ^2^ difference test were used to inform model fit when exploring more parsimonious models.

## Results

### Phenotypic results

Descriptive statistics, sex and age effects for all variables are given in Table 1. Panic/agoraphobia, separation anxiety symptoms, social anxiety, physical injury fears, generalised anxiety symptoms and hyperactivity/impulsivity were significantly higher in females than in males.

**Table 1:** Descriptive statistics for study variables and tests for sex and age effects

	Whole sample Mean (*SD*) (*n* = 1564)	Males Mean (*SD*) (*n* = 649)	Females Mean (*SD*) (*n* = 915)	Min‐Max	Regression coefficients (CIs)	
					Sex	Age
Panic/agoraphobia	1.39 (2.23)	1.11 (1.93)	1.58 (2.39)	0–18	.21 (.11, .31)^a^	−.03 (−.07, .01)
Separation anxiety symptoms	2.72 (1.42)	2.35 (1.18)	2.97 (1.51)	0–9	.43 (.33, .54)^a^	−.01 (−.06, .03)
Social anxiety	4.38 (2.69)	3.68 (2.47)	4.84 (2.73)	0–12	.43 (.32, .53)^a^	−.02 (−.03, .06)
Physical injury fears	3.03 (2.51)	2.10 (2.03)	3.64 (2.61)	0–15	.61 (.51, .71)^a^	−.01 (−.07, .02)
Obsessive‐compulsive symptoms	3.06 (2.93)	3.05 (2.90)	3.07 (2.94)	0–18	.01 (−.01, .11)	−.01 (−.05, .02)
Generalised anxiety symptoms	4.87 (2.91)	4.12 (2.73)	5.36 (2.92)	0–18	.42 (.32, .53)^a^	.00 (−.04, .04)
Attention problems	2.98 (2.19)	2.93 (2.21)	3.01 (2.17)	0–12	.04 (−.07, .14)	.00 (−.05, .04)
Hyperactivity/impulsivity	1.91 (1.56)	1.67 (1.46)	2.06 (1.61)	0–6	.25 (.15, .36)^a^	−.02 (−.06, .02)

Means and standard deviations are on raw (nonregressed) data to be maximally relevant to the reader.

Sex and age effects were evaluated with linear regression models on standardised values using the cluster command to account for the nonindependence of observations (e.g. twin/sibling pairs).

a Effect of sex at *p* < .001 level of significance representing higher scores in females (males = 0, females = 1).

Phenotypic correlations (Table 2) showed moderate associations between anxiety subtypes and attention problems, with the exception of separation anxiety (for which the association was nonsignificant). Conversely, the correlations between anxiety subtypes and hyperactivity/impulsivity problems were all much lower, especially for social anxiety. Partial correlations between anxiety subtypes and attention problems remained moderate when controlling for hyperactivity/impulsivity (Table 2). Conversely, correlations between anxiety subtypes and hyperactivity/impulsivity became nonsignificant or negative when controlling for attention problems. Of note, the negative association between social anxiety and hyperactivity/impulsivity was greater in magnitude when controlling for attention problems. As the attention problems component appeared to be driving the relationship between ADH problems and anxiety subtypes, hyperactivity/impulsivity problems were excluded from genetic analyses. Furthermore, because separation anxiety was not associated with attention problems or hyperactivity/impulsivity problems, it was also excluded from genetic analyses. Hence, multivariate genetic analyses were conducted on the relationship between attention problems and panic/agoraphobia, social anxiety, physical injury fears, obsessive‐compulsive symptoms and generalised anxiety symptoms.

**Table 2:** Full correlations between the two ADH dimensions and anxiety subtypes, alongside partial correlations controlling for one ADH dimension at a time (95% confidence intervals)

	Full correlations		Partial correlations	
	AP	HYP	AP (control HYP)^a^	HYP (control AP)^b^
Panic/agoraphobia	.39 (.35, .44)	.16 (.12, .22)	.34 (.30, .39)	.04 (−.02, .09)
Separation anxiety symptoms	−.05 (−.10, 0)	.02 (−.03, .07)	−.05 (−.10, 0)	.02 (−.03, .07)
Social anxiety	.33 (.28, .38)	−.04 (−.09, .01)	.36 (.31, .40)	−.19 (−.24, −.14)
Physical injury fears	.20 (.15, .25)	.09 (.04, .15)	.19 (.13, .24)	.03 (−.02, .08)
Obsessive‐compulsive symptoms	.41 (.36, .45)	.17 (.12, .22)	.34 (.29, .38)	.06 (.01, .11)
Generalised anxiety symptoms	.39 (.35, .44)	.17 (.12, .22)	.35 (.31, .39)	.03 (−.03, .08)
Attention problems	1	.32 (.27, .36)	1	–
Hyperactivity/impulsivity		1	–	1

AP, attention problems; HYP, hyperactivity/impulsivity.

a Partial correlation between attention problems and anxiety subtypes controlling for hyperactivity/impulsivity problems.

b Partial correlation between hyperactivity/impulsivity problems and anxiety subtypes controlling for attention problems.

### Genetic results

Twin correlations are shown in Table 3. For all variables MZ correlations were higher than DZ/sibling correlations, suggesting the presence of genetic effects. Multivariate models were fitted with scalars to take into account variance differences between males and females on anxiety scales detected when running univariate models (see Table S2). Results of the ACE correlated factor solution showed that shared environmental influences were all nonsignificant, and thus were dropped. The constrained AE model was not significantly different from the full ACE model (*χ* ^2 ^= 23130.77, *df* = 9148, AIC = 4834.77) and provided the best fit to the data (*χ* ^2 ^= 23135.35, *df* = 9169, *p* = 1.00, AIC = 4797.35).

**Table 3:** Twin correlations (95% confidence intervals) for study variables by zygosity

	MZm	MZf	DZm/MMsibs	DZf/FFsibs	DZo/OSsibs
Panic/agoraphobia	.53 (.35, .67)	.32 (.15, .47)	.12 (−.10, .31)	.18 (.03, .32)	.03 (−.10, .16)
Social Anxiety	.43 (.24, .59)	.34 (.17, .48)	.37 (.18, .54)	.24 (.09, .38)	.09 (−.04, .21)
Physical Injury Fears	.21 (.01, .41)	.41 (.25, .54)	.15 (−.05, .34)	.17 (.02, .31)	.07 (−.06, .19)
Obsessive‐compulsive Symptoms	.36 (.16, .53)	.45 (.30, .58)	.21 (−.01, .41)	.08 (−.08, .23)	.16 (.04, .28)
Generalised Anxiety Symptoms	.41 (.21, .57)	.48 (.33, .60)	.11 (−.11, .31)	.24 (.08, .39)	.10 (−.03, .23)
Attention Problems	.39 (.16, .60)	.44 (.28, .57)	.14 (−.08, .34)	.22 (.06, .36)	.26 (.13, .38)

MZm, monozygotic male; MZf, monozygotic female; DZm/MMsibs, dizygotic male and male siblings; DZf/FFsibs, dizygotic female and female siblings; DZo/OSsibs, dizygotic opposite‐sex and opposite‐sex siblings.

In Table 4, parameter estimates from the AE multivariate model are given. Overall, genetic variance components were moderate for all variables (.32–.45), whereas the rest of the variance was explained by nonshared environmental factors. Attention problems showed moderate‐to‐high genetic correlations (.45–.58) with all anxiety scales with the exception of physical injury fears, for which the genetic correlation was lower (.12) and not significant. Nonshared environmental correlations among anxiety subtypes and attention problems were all moderate (.21–.47). In addition, we estimated the proportion of a phenotypic correlation explained by overlapping A, C or and E by multiplying the square root of the variance component of each variable by the relevant genetic, shared environmental or nonshared environmental correlation, and then dividing this by the overall phenotypic correlation. For example, the proportion of phenotypic correlation between panic/agoraphobia and attention problems explained by A was calculated as (√.32*.53*√.45)/.39. Half or more of the phenotypic correlations between anxiety subtypes and attention problems were attributable to genetic influences (.50–.58), except for the association between physical injury fears and attention problems, which was explained to a greater extent (.78) by overlapping nonshared environmental influences.

**Table 4:** Parameter estimates (95% confidence intervals) from multivariate modelling: genetic and nonshared environmental influences on each variable are given in bold on the diagonal, genetic and nonshared environmental correlations are given above the diagonal, and proportions of correlations attributable to genes and nonshared environment are given below the diagonal

	PAN	SOC	PHY	OCS	GAS	AP
*Genetic influences*						
Panic/agoraphobia	**.32 (.22, .42)**	.32 (.10, .51)	.20 (−.06, .41)	.73 (.57, .89)	.68 (.53, .81)	.53 (.35, .70)
Social anxiety	.33 (.10, .55)	**.35 (.26, .44)**	.30 (.08, .49)	.52 (.35, .67)	.61 (.46, .75)	.45 (.28, .61)
Physical injury fears	.21 (−.05, .44)	.33 (.08, .56)	**.33 (.22, .43)**	.49 (.29, .67)	.25 (.03, .44)	.12 (−.09, .31)
Obsessive‐compulsive symptoms	.54 (.38, .70)	.46 (.28, .62)	.55 (.31, .77)	**.36 (.27, .45)**	.71 (.59, .83)	.58 (.43, .73)
Generalised anxiety symptoms	.45 (.30, .59)	.44 (.30, .57)	.30 (.04, .54)	.49 (.35, .61)	**.41 (.31, .50)**	.51 (.35, .65)
Attention problems	.50 (.31, .68)	.54 (.33, .73)	.22 (−.18, .55)	.58 (.40, .74)	.55 (.36, .71)	**.45 (.35, .53)**
*Non‐shared environmental influences*						
Panic/agoraphobia	**.68 (.58, .78)**	.33 (.23, .42)	.37 (.27, .46)	.32 (.22, .41)	.46 (.38, .55)	.32 (.21, .42)
Social anxiety	.67 (.45, .90)	**.65 (.56, .74)**	.31 (.22, .41)	.34 (.25, .43)	.47 (.38, .55)	.26 (.15, .36)
Physical injury fears	.79 (.56, 1)	.67 (.44, .92)	**.67 (.57, .78)**	.21 (.11, .31)	.34 (.23, .43)	.27 (.16, .37)
Obsessive‐compulsive symptoms	.46 (.30, .62)	.54 (.38, .72)	.45 (.23, .69)	**.63 (.55, .73)**	.47 (.38, .55)	.28 (.18, .38)
Generalised anxiety symptoms	.55 (.41, .70)	.56 (.43, .70)	.70 (.46, .96)	.51 (.39, .65)	**.59 (.50, .69)**	.32 (.21, .42)
Attention problems	.50 (.32, .69)	.46 (.27, .67)	.78 (.45, 1)	.42 (.26, .60)	.45 (.29, .64)	**.55 (.47, .65)**

Confidence intervals not spanning zero indicate significance.

PAN, panic/agoraphobia; SOC, social anxiety; PHY, physical injury fears; OCS, obsessive‐compulsive symptoms; GAS, generalised anxiety symptoms; AP, attention problems.

## Discussion

The present study adds to our understanding of the relationship between anxiety and ADHD in two novel ways. First, by disaggregating these phenotypes into subtypes, we found that associations between anxiety subtypes and ADH dimensions were attributable to attention problems, and not to hyperactivity/impulsivity. Second, by examining the aetiological architecture of significant phenotypic associations, we showed that aetiological overlap between attention problems and anxiety subtypes involved both genetic and nonshared environmental influences, although patterns varied between pairs of symptoms.^1^

A number of previous studies, using both clinical and population‐based samples, have reported an association between anxiety and ADHD (Biederman et al., 1991; Bowen et al., 2008). However, the phenotypic relationships between different dimensions of ADH symptoms and anxiety have been scarcely explored. In our study, separating the two ADH dimensions, we found moderate associations between attention problems and the majority of anxiety subtypes, whereas the associations between hyperactivity/impulsivity and anxiety subtypes were predominantly small and/or negligible. This would suggest that the association between anxiety and ADH problems is driven by the association between anxiety and attention problems, but not hyperactivity/impulsivity. Of note, partial correlations showed that social anxiety had a significant negative correlation with hyperactivity/impulsivity but not with attention problems. This may indicate that the social anxiety subtype selectively inhibits symptoms of hyperactivity/impulsivity only (and/or vice‐versa), or that shared aetiological factors have an effect on both conditions (twin correlations and genetic model‐fitting analyses on this association are reported in Table S3 and Table S4 respectively). This finding is consistent with data from studies reporting that anxiety is associated with a suppression of hyperactive behaviour, but not inattention, in ADHD youth (Epstein et al., 1997; Pliszka, 1992).

Attention problems were moderately associated with panic/agoraphobia, social anxiety, obsessive‐compulsive symptoms and generalised anxiety, whereas the association with physical injury fears was weaker and that with separation anxiety nonsignificant. This is partially consistent with a previous study on a clinical sample of children with ADHD, which found that the anxiety diagnoses most often co‐occurring with ADHD were generalised anxiety disorder, specific phobias, obsessive‐compulsive disorder, social anxiety and separation anxiety disorder (Vance, Harris, Boots, Talbot, & Karamitsios, 2003). In the current study, separation anxiety symptoms were not associated with ADH dimensions. This may reflect the mean age of our late adolescent sample, given that separation anxiety is typically recognised as a disorder of childhood (Beesdo, Knappe, & Pine, 2009; Lewinsohn, Holm‐Denoma, Small, Seeley, & Joiner, 2008). Overall, phenotypic findings may be interpreted in light of the cognitive profiles of inattentive and anxious individuals. Anxiety is characterised by enhanced levels of attention towards threat stimuli (Beck & Clark, 1997), which leads to reduced attentional resources for other stimuli (Cisler & Koster, 2010). For example, the focus of attention in individuals with high levels of panic/agoraphobia may be shifted towards their physical symptoms and the worry of future panic attacks, which may in turn result in attention deficits in everyday tasks. Similar reasoning might be applied to other anxiety subtypes, whereby anxious people experience attention problems as a consequence of their attention biases towards threat stimuli.

In the present study, genetic influences on each variable were slightly lower than environmental effects. For anxiety, this pattern fits with the results of previous studies (Eley et al., 2003; Ogliari et al., 2010). Conversely, the heritability of attention problems is typically reported as being higher than we report – in the region of .70 (Greven et al., 2011; McLoughlin et al., 2007; Moruzzi, Rijsdijk, & Battaglia, 2014; Schmitz & Mrazek, 2001). However, where most twin studies examining ADH problems have used parent‐report measures on younger participants, in our late adolescent sample we have used self‐report, which frequently results in lower heritability estimates when compared to those derived from parent ratings (Chang, Lichtenstein, Asherson, & Larsson, 2013; Merwood et al., 2013). As such, our lower heritability estimate should not be viewed as unusual and indeed aligns with other studies using self‐report measures (Chang et al., 2013; Larsson et al., 2013; Merwood et al., 2013).

Prior to our study, only a few researchers had examined the aetiological overlap underlying the association between anxiety and ADH problems, and none had considered their heterogeneity. Genes and the nonshared environment, but not the shared environment, were found to play a significant role in the covariation between variables. With the exception of physical injury fears, genetic correlations between anxiety symptoms and attention problems were around .50. These estimates are lower than those found in the only previous twin study in the literature (Schmitz & Mrazek, 2001) exploring the association of attention problems and CBCL internalising scales in a small sample of children aged 4–11 (.71 with ‘anxious/depressed’ and .79 with ‘withdrawn’ scales). The lower genetic correlations may be due to the different age range covered by our adolescent sample compared to this previous study. Alternatively, our use of self‐report measures, as opposed to Schmitz & Mrazek's use of parent‐report measures, may explain the difference. Further research is needed to clarify this issue.

With the exception of the relationship between physical injury fears and attention problems, phenotypic correlations between anxiety subtypes and attention problems were approximately 50% attributable to genetic overlap. These results contrast somewhat with the observation that the nonshared environment accounted for more variance in our variables than did genetic factors. This pattern of results, whereby unique variance in phenotypes is predominantly of nonshared environmental origin, whereas covariance between them is genetic, aligns with the idea that the relationship between disorders might be attributable to common genes, while differences are attributable to environmental factors (Eley, 1997; Kovas & Plomin, 2006). The only anxiety subtype showing a different pattern of results was physical injury fears, for which relationships with other anxiety subtypes and attention problems were primarily explained by overlap in nonshared environment influences. This is consistent with previous studies showing that the genetic aetiology of specific phobias might be distinct from that of other anxiety disorders (Hettema, Prescott, Myers, Neale, & Kendler, 2005; Kendler, Prescott, Myers, & Neale, 2003). There results highlight that future evaluations of the phenotypic and aetiological association between adolescent anxiety and ADH problems should therefore take into account their symptom dimensions, as has been previously argued with respect to child anxiety (Eley et al., 2003).

The genetically informative sample and the distinction between subtypes of anxiety and ADHD dimensions are clear strengths of the current study. However, some limitations should be acknowledged. Self‐report questionnaires were used, which may only partially capture the complexity of anxiety and ADH problems. Additionally, both ADH dimension derived with the factor analysis (6 for attention problems, 3 for hyperactivity/impulsivity) consisted of fewer items than those included for each ADHD dimension included in DSM‐5 (American Psychiatric Association, 2013). Although Cronbach's alphas demonstrated acceptable levels of internal validity, the low number of items may have prevented a thorough measure of ADH problems, and affected the pattern of phenotypic and genetic relationship with anxiety. Also, since this is a general population sample, results may not generalise to more severe or ‘clinical’ individuals. However, it has been shown that heritability estimates obtained using dichotomous measures of psychiatric disorders are comparable to those obtained using continuous, normally distributed measures of psychopathology (Larsson, Anckarsater, Rastam, Chang, & Lichtenstein, 2012; Plomin, Haworth, & Davis, 2009). As such, there is no clear evidence to suggest our findings would not generalise to studies using psychiatric diagnoses of ADHD and anxiety disorders.

## Conclusions

Our results show that the associations between anxiety and ADH dimensions are likely to be primarily due to an association between anxiety and attention problems, rather than between anxiety and hyperactivity/impulsivity. Additionally, exploring the aetiological sources of associations, we found that these relationships were explained by shared genetic and nonshared environmental factors. Our findings suggest that future studies of psychiatric comorbidity should disaggregate anxiety and ADH problems into their subtypes, taking into account the phenotypic and aetiological heterogeneity within each disorder (e.g. ADHD) and broad disorder categories (e.g. anxiety disorders). More generally, it is clear that the study of anxiety and ADHD comorbidity is complex and warrants further research. Given that anxiety and ADH problems frequently co‐occur, and that this comorbidity is partly explained by genes and partly by environmental factors, future studies should expand these results investigating the mechanisms underlying the genetic and environmental overlap between ADH problems and anxiety subtypes over time. In particular, longitudinal designs may successfully explore the temporal continuity of genetic and environmental influences on anxiety and ADH dimensions, and examine whether this co‐occurrence is entirely explained by the same factors acting simultaneously, or whether factors influencing anxiety lead to ADH problems or vice‐versa. In turn, this may aid in the development of specific treatment and prevention strategies for individuals with comorbid ADHD and anxiety disorders.

**Key points**

- Anxiety and ADHD symptoms are highly heterogeneous and often co‐occur in adolescence.
- Little is known about their phenotypic and aetiological association.
- In this study the association between ADH problems and anxiety can be attributed to the attention problems component of ADHD, not hyperactivity/impulsivity.
- A considerable proportion of most of the correlations between anxiety subtypes and attention problems is due to substantial genetic overlap.
- High heterogeneity within disorders should be considered to better understand the nature of co‐occurrence between disorders.

## Supporting information


**Table S1.** Loadings for attention problems and hyperactivity/impulsivity problems.
**Table S2.** Univariate fit statistics and genetic and environmental estimates.
**Table S3.** Twin correlations for social anxiety and hyperactivity/impulsivity.
**Table S4.** Parameter estimates from bivariate modelling between social anxiety and hyperactivity/impulsivity.

## Note

1 The overlap between anxiety subtypes was not the focus of this study and is not therefore discussed in detail. Phenotypic and genetic overlap between anxiety subtypes in this sample are described elsewhere (Lau et al., 2007; Waszczuk, Zavos, Gregory, & Eley, 2014).
