# Supplementary material for: Aetiological overlap between anxiety and attention deficit hyperactivity symptom dimensions in adolescence
Source: J Child Psychol Psychiatry. 2014 Sep 8;56(4):423–31. doi: 10.1111/jcpp.12318 (PMC6607691; doi:10.1111/jcpp.12318)
Supplement: Supplementary file 1 — Table S1. Loadings for attention problems and hyperactivity/impulsivity problems. Table S2. Univariate fit statistics and genetic and environmental estimates. Table S3. Twin correlations for social anxiety and hyperactivity/impulsivity. Table S4. Parameter estimates from bivariate modelling between social anxiety and hyperactivity/impulsivity. [file JCPP-56-423-s001.docx]

***Online supplementary material for: Aetiological overlap between anxiety and attention deficit hyperactivity symptom dimensions in adolescence; by Michelini et al***

[**Table S1**. Loadings for attention problems and hyperactivity/impulsivity problems](#tableS1)

[**Table S2.** Univariate fit statistics and genetic and environmental estimates](#tableS2)

[**Table S3.** Twin correlations for social anxiety and hyperactivity/impulsivity](#tableS3)

[**Table S4.** Parameter estimates from bivariate modelling between social anxiety and hyperactivity/impulsivity](#tableS4)

**Table S1.** Loadings for attention problems (Factor 1) and hyperactivity/impulsivity problems (Factor 2) in an exploratory factor analysis of ADH dimensions.

| **Items** | **Factor 1** | **Factor 2** |
| --- | --- | --- |
| **I act too young*** | .35 | .22 |
| **I day-dream a lot** | **.59** | .02 |
| **I have trouble to concentrate** | **.74** | .13 |
| **I talk too much** | .03 | **.79** |
| **I am louder than others** | -.02 | **.83** |
| **I have trouble standing still** | .30 | **.60** |
| **I act without stopping to think*** | .47 | .45 |
| **I fail to finish things** | **.67** | .13 |
| **My behaviour is irresponsible** | **.58** | .26 |
| **I am too dependent** | **.57** | -.01 |
| **My school work or job performance is poor** | **.60** | .09 |

***** *Cross-loading items not included in* *attention problems and hyperactivity/impulsivity problems scales for analyses.*

*Note: exploratory factor analysis was repeated on the other half of the sample and led to the same two factors (results available from first author upon request).*

**Table S2.** Univariate fit statistics and genetic and environmental estimates for attention problems and anxiety subtypes (95% confidence intervals).

|  | **Fit statistics** | | | **Standardised variance components** | | |
| --- | --- | --- | --- | --- | --- | --- |
|  | **-2LL** | **df** | **AIC** | **A** | **C** | **E** |
| **Panic/agoraphobia** | 4219.45 | 1537 | 1145.45 | .35 (.18-.45) | 0 (0-.11) | .65 (.55-.76) |
| **Social anxiety** | 4278.51 | 1542 | 1194.51 | .29 (0-.45) | .06 (0-.28) | .65 (.55-.77) |
| **Physical injury fears** | 4347.28 | 1562 | 1223.38 | .34 (.12-.44) | 0 (0-.15) | .66 (.56-.78) |
| **Obsessive-compulsive symptoms** | 4330.02 | 1551 | 1228.02 | .36 (.16-.45) | 0 (0-.14) | .64 (.55-.74) |
| **Generalised anxiety symptoms** | 4264.1 | 1529 | 1206.1 | .41 (.24-.50) | 0 (0-.12) | .59 (.50-.69) |
| **Attention problems** | 4059.6 | 1471 | 1117.6 | .43 (.14-.53) | .01 (0-.21) | .56 (.47-.68) |

*A – additive genetic influences, C - shared environmental influences, E – nonshared environmental influences.*

*Note: univariate genetic analyses involved exploring quantitative and qualitative sex differences for all variables. Results showed no such sex differences for any of the variables, although variance differences were found between males and females for panic/agoraphobia, physical injury fears, obsessive-compulsive symptoms, generalised anxiety symptoms (with females showing higher variability). Univariate models for these variables and multivariate models were therefore fitted including scalars to account for these variance differences.*

**Table S3.** Twin correlations (95% confidence intervals) for social anxiety and hyperactivity/impulsivity (after regressing out attention problems from both scales) by zygosity.

|  | **MZm** | **MZf** | **DZm/MMsibs** | **DZf/FFsibs** | **DZo/OSsibs** |
| --- | --- | --- | --- | --- | --- |
| **Social anxiety (controlling for Attention Problems)** | .44 (.23,.63) | .32 (.15,.47) | . 32 (.11,.51) | .23 (.07,.38) | .07 (-.07,.21) |
| **Hyperactivity/impulsivity (controlling for Attention Problems)** | .45 (.25,.63) | .55 (.41,.66) | -.02 (-.25,.21) | .07 (-.08,.22) | .01 (-.14,.12) |

*MZm – Monozygotic male, MZf – Monozygotic female, DZm/MMsibs – Dizygotic male and male siblings, DZf/FFsibs – Dizygotic female and female siblings, DZo/OSsibs – Dizygotic opposite-sex and opposite-sex siblings.*

**Table S4.** Parameter estimates (95% confidence intervals) from bivariate modelling between social anxiety and hyperactivity/impulsivity (after regressing out attention problems from both scales): genetic and non-shared environmental influences on each variable are given in bold on the diagonal, genetic and non-shared environmental correlations are given above the diagonal, and proportions of correlations attributable to genes and non-shared environment are given below the diagonal.

|  | **Genetic influences** | | **Non-shared environmental influences** | |
| --- | --- | --- | --- | --- |
|  | **SOC** | **HYP** | **SOC** | **HYP** |
| **Social anxiety**  **Hyperactivity/impulsivity** | **.34 (.24,.44)**  .60 (.19,.98) | -.32 (-.53,-.10)  **.38 (.27,.48)** | **.66 (.56,.76)**  .40 (.02,.80) | -.12 (-.23,-.01)  **.62 (.52,.73)** |

*SOC – Social anxiety, HYP – Hyperactivity/impulsivity. Confidence intervals not spanning zero indicate significance.*

*Note: Twin correlations for social anxiety showed that MZ correlations were less than twice DZ correlations, suggesting shared environmental effects, whereas MZ correlations for hyperactivity/impulsivity were more than twice DZ correlations, suggesting non-additive genetic effects. We fitted two bivariate models, one with ACE parameters estimated for each variable, and the other with ADE estimated for each. Both models could be constrained to an AE model (χ^2^=8133.80, df=2926, AIC=2281.79). This enabled us to estimate the degree of overlap between the aetiological factors influencing the two variables and the proportions of phenotypic correlation explained by overlapping genes and non-environmental influences. The negative genetic correlation between the two variables indicates that a moderate proportion of the genes which are associated with increased levels of symptoms on one phenotype are associated with decreased levels of symptoms on the other phenotype.*
